# Infectious intracranial aneurysm associated with *Lactococcus garvieae*: A case report and literature review

**DOI:** 10.1016/j.imj.2024.100123

**Published:** 2024-07-30

**Authors:** Chung-Ho Lee, Peter Yat-Ming Woo, Calvin Ka-Lam Leung, Ronald Li, Jenny Kwan-Tsz Chan, Kwan-Shun Ng, Cindy Wing-Sze Tse

**Affiliations:** a Department of Pathology, Kwong Wah Hospital, Hong Kong Special Administrative Region, China; b Department of Neurosurgery, Kwong Wah Hospital, Hong Kong Special Administrative Region, China

**Keywords:** *Lactococcus garvieae*, Bacteraemia, Infectious intracranial aneurysm, Mycotic aneurysm, Subarachnoid haemorrhage

## Abstract

*Lactococcus garvieae* is a known fish pathogen associated with numerous aquacultural outbreaks. In humans, *L. garvieae* primarily causes infective endocarditis, but infections involving other organs have also been reported. We report the first case of ruptured infectious intracranial aneurysm associated with *L. garvieae* bacteraemia without concomitant infective endocarditis. The diagnosis of a left distal posterior cerebral artery mycotic aneurysm was based on a computed tomography angiogram, catheter angiogram and histopathological examination of the resected aneurysm. Here, we review the literature on human *L. garvieae* infections and describe the clinical characteristics, risk factors, management and outcomes of the cases identified to date.

## Highlights

• *Lactococcus garvieae* is a known fish pathogen. Its role as an emerging zoonotic pathogen is increasingly recognised as more cases are reported.
• Infective endocarditis (both native and prosthetic valve) accounts for the majority of human *L. garvieae* infections, but infections of other organ systems and contamination of platelet concentrates have also been reported.
• The risk factors associated with *L. garvieae* infection include raw fish contact or consumption and underlying gastrointestinal diseases. Exposure to raw fish was found in one-third of the reported cases of *L. garvieae* infection, while around half of the reported cases had underlying gastrointestinal disorders.
• β-lactams are the drug of choice for infections due to *L. garvieae*, clindamycin should be avoided as the organism is intrinsically resistant.
• There were two cases of infectious intracranial aneurysm due to *L. garvieae* infection reported in the literature, but both cases could be attributed to the underlying infective endocarditis. We report the first case of a ruptured infectious intracranial aneurysm in a patient with *L. garvieae* bacteraemia without concomitant endocarditis.

## 1 Case report

A 59-year-old man, who had undergone mechanical aortic valve replacement 25 years ago for aortic stenosis, experienced severe acute onset occipital headache for five days associated with dizziness, neck pain and vomiting. There was no history of head injury. The patient was afebrile and conscious upon admission. There was nuchal rigidity but no focal neurological deficit.

**Fig. 1:**
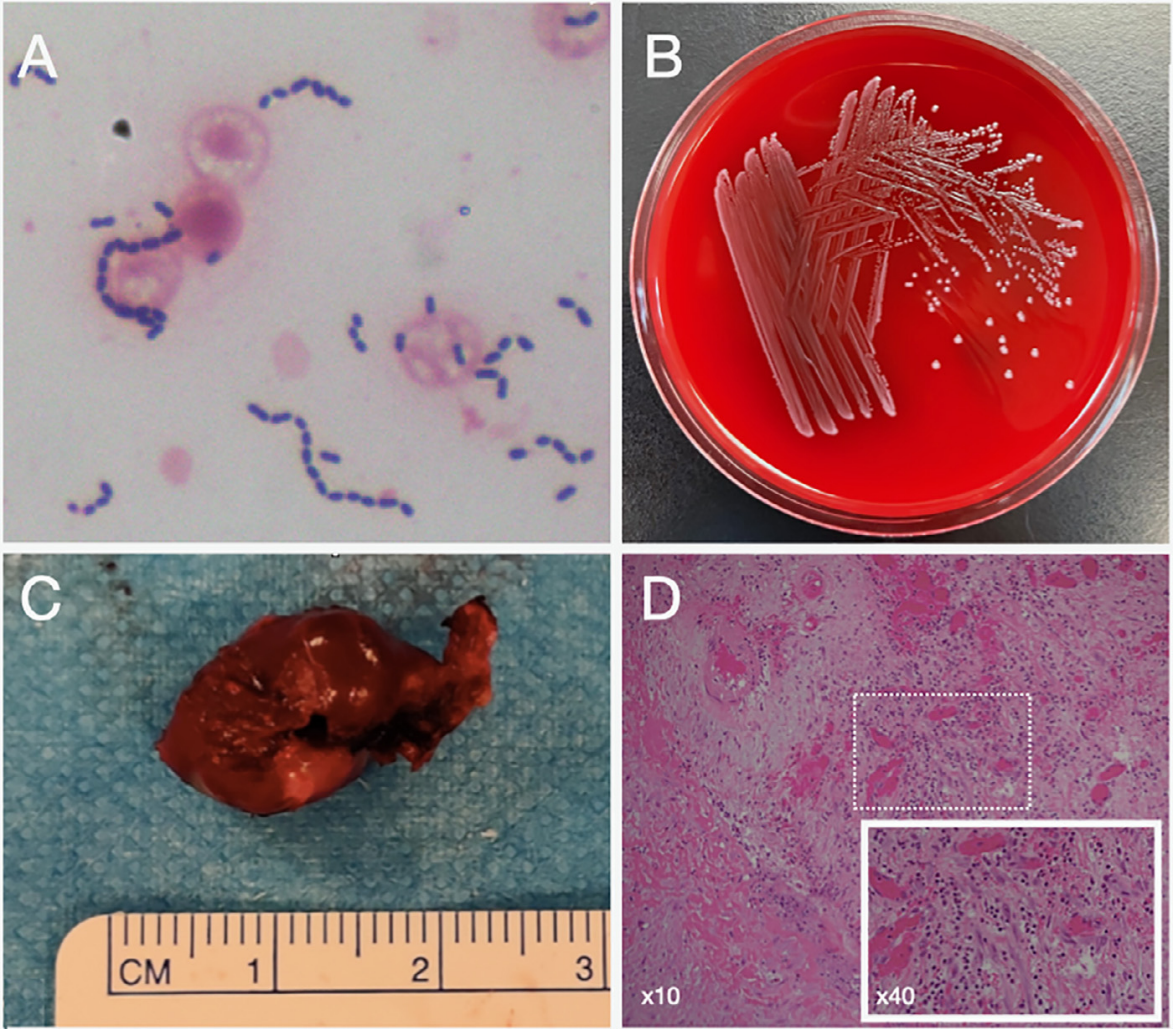
Gram stain of the positive blood culture showing Gram-positive cocci in chains (A). Colony morphology of *L. garvieae* (B). Gross appearance of the aneurysm (C). Haematoxylin and eosin staining demonstrating the aneurysm wall with mixed neutrophilic and lymphocytic infiltrates, magnification 100×. Inset magnification 400× (D).

His complete blood count was significant for microcytic hypochromic anaemia and neutrophil-predominant leukocytosis. Liver and renal function tests were unremarkable. Blood culture taken on admission was positive for Gram-positive cocci in chains after one day of incubation in the BD BACTEC™ FX blood culture system (Fig. 1A and B). The organism was identified as *Lactococcus garvieae* by matrix-assisted laser desorption ionization-time of flight mass spectrometry with Bruker Microflex® LT (Bruker Daltonics, Bremen, Germany) and automated biochemical testing system with VITEK 2 (bioMérieux, Marcy l'Etoile, France). The isolate was sensitive to penicillin, ceftriaxone and vancomycin according to the Clinical Laboratory and Standards Institute breakpoints. The minimum inhibitory concentrations of penicillin, ceftriaxone and vancomycin were 0.19 µg/mL, 0.064 µg/mL and 0.5 µg/mL, respectively.

**Fig. 2:**
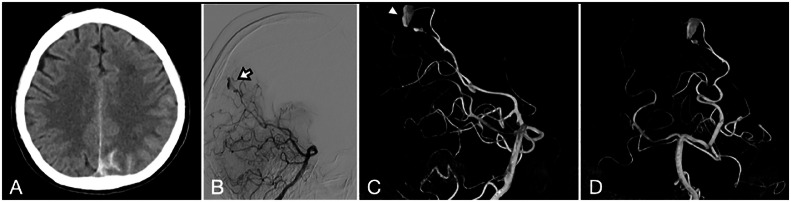
Axial non-contrast-enhanced CT head scan revealing subarachnoid haemorrhage in the left parieto-occipital region (A). Catheter angiogram after two-weeks of intravenous antibiotics demonstrating an enlarged left distal posterior cerebral artery aneurysm (B, white arrow, left vertebral artery injection lateral view; C, white arrowhead, AP view 3D reconstruction; D, lateral view 3D reconstruction).

Non-contrast computed tomography (CT) of the brain showed acute subarachnoid haemorrhage in the left parieto-occipital region (Fig. 2A). A CT angiogram detected a 12 mm distal posterior cerebral artery (PCA) fusiform aneurysm at the praecuneus. Three additional sets of blood culture from separate sites were all positive for *L. garvieae* after one day of incubation. The patient was managed with intravenous ceftriaxone (1 g Q12H), which resulted in clearance of the bacteraemia.. A transthoracic and transoesophageal echocardiogram revealed no evidence of infective endocarditis (IE). There were also no evidence of other embolic or immunological phenomena.

In view of the *L. garvieae* bacteraemia, history regarding fish exposure was explored. The patient consumed a takeaway assorted sushi and sashimi platter with his family four days prior to the onset of his headache and developed diarrhoea two days afterwards, which subsided before hospitalisation. He had no history of undercooked food intake otherwise. His family members remained asymptomatic.

After two weeks of antibiotics, the patient's headache subsided, and serial CT scans showed resolution of the subarachnoid haemorrhage. However, a catheter angiogram revealed an expansion of the distal PCA aneurysm to a diameter of 14 mm (Fig. 2B–D). In view of the aneurysm's enlargement despite antibiotics, a craniotomy for its resection was performed. Histopathological examination of the aneurysm tissue demonstrated mixed neutrophilic and lymphocytic infiltrates compatible with an infected aneurysm (Fig. 1C and D). The patient was discharged after completing six weeks of ceftriaxone. A screening contrast-enhanced CT of the abdomen and pelvis and colonoscopy were performed. The CT scan was unremarkable, but two tubular adenomas at the caecum and transverse colon were identified by colonoscopy and excised. One year after the subarachnoid haemorrhage, the patient remained well with a modified Rankin score of 1.

## 2 Discussion

*L. garvieae* is a Gram-positive, facultatively anaerobic organism appearing as cocci in chains. It is known to cause lactococcosis, an acute septicaemic syndrome affecting multiple marine and freshwater animal species [1,2]. Owing to a high mortality rate of up to 50%, lactococcosis outbreaks can cause significant economic losses in the aquaculture industry. Apart from aquatic organisms, *L. garvieae* has been isolated from a multitude of foods including milk, cheese, meat and fish products [1]. The potential virulence factors include haemolysin III-related proteins, siderophores, internalin, mucus adhesin and sortase A [1].

The role of *L. garvieae* as an emerging zoonotic pathogen is increasingly recognised with 57 cases reported. The majority of *L. garvieae* infections (54.4%, 31/57) are IE (Table 1). For *L. garvieae* IE, both sexes are affected; the male-to-female ratio of the reported cases is 1.4:1. The median age of affected patients is 68 (interquartile range: 59.0–77.5). Nearly half of the patients affected (48.4%, 15/31) had prosthetic valve IE and fatal infections occurred in five cases (16.1%). All patients were treated with systemic antibiotics and around one-third (11/31) of the patients required valve replacement.

**Table 1:** Published literature on cases of infective endocarditis caused by *Lactococcus garvieae*.

Reference	Sex/ Age	Comorbid conditions	Site	Clinical presentation	Fish contact/ consumption	Complications	Treatment	Outcome
Rösch et al. [7]	M/78	Atrial fibrillation, hypertension, chronic kidney disease, right external carotid artery stenosis, history of stenting of right coronary artery	Prosthetic aortic valve	Fever, exertional dyspnoea, expectoration, aortic systolic murmur	No	Nil	Aortic valve replacement. 6 weeks of penicillin and 2 weeks of gentamicin	Survived
Zuily et al. [15]	F/64	Atrial fibrillation, chronic hepatitis C with cirrhosis, history of pacemaker implantation	Prosthetic mitral valve	Fever	Yes (consumption of fresh seafood)	Nil	Amoxicillin and gentamicin for 6 weeks	Survived
Malek *.* [6]	M/50	Chronic rheumatic heart disease with mitral valve replacement, dislipidaemia, reflux oesophagitis	Native aortic valve	Intracranial haemorrhage, flu-like symptoms	No	Intracranial haemorrhage	Ceftriaxone for 6 weeks and gentamicin for 2 weeks. Aortic valve replacement	Survived
Ortiz et al. [16]	F/70	None	Native mitral valve	Progressive heart failure	No	Heart failure	Vancomycin for 6 weeks. Mitral valve replacement	Survived
Ortiz et al. [16]	F/77	Hypertension, chronic lymphocytic leukaemia, colorectal cancer with surgery done	Native aortic and mitral valves	Purpuric lesions, fever, acute renal failure	No	Heart failure	Ampicillin and gentamicin	Died
Russo et al. [17]	M/63	Type A aortic dissection with grafting of aorta and aortic valve replacement, abdominal aortic aneurysm, mitral insufficiency with repair using autologous pericardium, colonic diverticulosis	Native mitral valve	Fever with chills, pharyngodynia and generalised weakness	Yes (consumption of Italian naturally fermented cheese and fish)	Nil	Vancomycin and gentamicin for 2 weeks, followed by ampicillin for 2 weeks	Survived
Lim *et al.* [18]	M/85	Erosive gastritis, aortic valve replacement, history of tricuspid valve infective endocarditis	Native mitral valve	Anorexia, right lower limb cramps, acute urinary retention	No	Embolic stroke causing central post-stroke pain syndrome	Ceftriaxone for 6 weeks	Survived
Watanabe et al. [12]	F/55	Nil	Native mitral valve	Fever, malaise and myalgia	No	Renal infarction, cerebral infarction, and cerebral mycotic aneurysms	Ceftriaxone and gentamicin	Survived
Heras et al. [19]	M/68	Hypertension, dyslipidemia, Hodgkin lymphoma in remission, complete atrioventricular block with pacemaker implantation, aortic stenosis with aortic valve replacement	Native mitral valve	Fever	No	Mitral abscess, heart failure	Daptomycin, ampicillin, ceftriaxone and gentamicin	Died
Hirakawa et al. [20]	F/58	Hypertension, type 2 diabetes mellitus, dyslipidaemia, mitral stenosis with mitral valve replacement	Prosthetic mitral valve	Fever, chills, diaphoresis, erythematous nodules in hands and legs, myalgia and weakness	Yes (Consumption of fish and white cheese, with gingival perforation due to fish bone)	Nil	Vancomycin	Survived
Tsur et al. [21]	M/76	Aortic stenosis with aortic valve replacement, congestive heart failure, atrial fibrillation, type 2 diabetes mellitus, essential hypertension, oesophageal adenocarcinoma with total oesophagectomy	Prosthetic aortic valve	Fever, constipation	Yes (Consumption of sushi)	Nil	Ceftriaxone and gentamicin	Survived
González-Bravo et al. [22]	M/78	Essential hypertension, dyslipidaemia, gastroesophageal reflux disease, atrial fibrillation, aortic valve replacement	Prosthetic aortic valve	Fever, chills, altered mental status, malaise, anorexia	Yes (Consumption of raw salmon)	Nil	Ceftriaxone for 6 weeks	Survived
Rasmussen et al. [23]	M/81	Myocardial infarction with coronary artery bypass grafting, stening of carotid artery, aortic valve replacement, atrial fibrillation, rectal diverticulosis	Native mitral valve, prosthetic aortic valve	Malaise, headache, expressive dysphasia	No	Left fronto-temporal subdural haematoma and left cerebellar hemisphere infarction	Penicillin for 4 weeks and tobramycin for 3 weeks	Survived
Wilbring et al. [24]	M/55	Tricuspid valve replacment	Prosthetic tricuspid valve	Progressive dyspnoea, fever, chills	Yes (Amateur fish farmer)	Nil	Levofloxacin, amoxicillin-clavulanate for 8 weeks, prosthetic tricuspid valve replacement	Survived
Fleming et al. [25]	M/68	Type 2 diabetes mellitus, non-Hodgkin lymphoma in remission, aortic valve replacement	Native mitral valve and prosthetic aortic valve	Fever, migratory arthralgia, functional decline, anorexia, fatigue, weight loss	Yes (Consumption of raw fish)	Nil	Vancomycin for 6 weeks	Died
Lim et al. [26]	M/57	Gallstones, colonic polyps	Native mitral valve	Weight loss, fever, and anaemia	No	Nil	Amoxicillin and gentamicin for 6 weeks. Mitral and aortic valve replacement (no vegetation over aortic valve, but replacment done in view of moderate-to-severe aortic regurgitation)	Survived
Li et al. [27]	M/41	None	Native mitral valve	Right hemiplegia and slurring of speech	Yes (Working as a chef with frequent raw fish contact)	Acute cerebral infarction over the left middle cerebral artery territory	Penicillin and gentamicin for 30 days, mitral valve replacement	Survived
Fihman et al. [28]	F/86	Aortic valve replacement, colonic diverticulosis, duodenal ulcer	Prosthetic aortic valve	Fever and right hip pain	No	Nil	Amoxicillin for 7 weeks and gentamicin for 3 weeks	Survived
Navas et al. [29]	M/64	Type 2 diabetes mellitus, hypertension, chronic obstructive pulmonary disease, mitral valve repair, coronary artery bypass grafting, intracardiac difibrillator insertion	Native aortic valve	Progressive fatigue, weight loss, and anorexia	No	Nil	Vancomycin for 6 weeks. Aortic valve replacement, ascending aorta repair, ICD lead and pacemaker removal	Survived
Fefer et al. [30]	F/84	Hypertrophic cardiomyopathy, complete heart block with pacemaker insertion, hypothyroidism, immune thrombocytopenic purpura, aortic valve replacement	Native mitral valve	Progressive exertional dyspnoea, generalised weakness, anorexia	No	Ruptured cordae tendinae, pulmonary oedema, haemorrhagic pleural effusion, massive left cerebral haemorrhage	Ceftriaxone. Mitral valve replacement	Died
Suh et al. [31]	F/75	Mitral stenosis with mitral valve replacment, gastritis	Prosthetic mitral valve and native aortic valve	Progressive heart failure	Yes (Consumption of raw fish)	Pulmonary oedema	Ceftriaxone for 26 days. Mitral and aortic valve replacement	Survived
Vinh et al. [32]	M/80	Type 2 diabetes mellitus, hypercholesterolaemia, coronary artery disease, malignant colonic polyps with resection done	Native aortic valve	Dypsnoea and epigastric discomfort	No	Congestive heart failure	Ampicillin for more than 6 weeks. Aortic valve replacement	Survived
Yiu et al. [33]	M/67	Chronic rheumatic heart disease, atrial fibrillation	Native mitral valve	Fever, chills and rigors	Yes (Frequent visits to fish market)	Partial rupture of chordae tendinae	Ampicillin for 6 weeks	Survived
Bazemore et al. [34]	M/45	Hepatitis C with cirrhosis, aortic root aneurysm with Bentall repair, familial adenomatous polyposis	Prosthetic aortic valve	Symptomatic anaemia, generalised malaise and weakness,	No	Splenic and left renal infarct. Near-complete dehiscence of the valve conduit from the aortic annulus and significant destruction of the aortic annulus with a large pseudoaneurysm	Ceftriaxone for 6 weeks and gentamicin for 2 weeks. Aortic root repair and aortic valve replacement	Survived
James et al. [35]	F/56	Aortic stenosis with aortic valve replacement	Possible prosthetic aortic valve	Low back pain, rigors, night sweats, anorexia, weight loss	No	Vertebral osteomyelitis	Teicoplanin for 2 months	Survived
Backes et al. [36]	F/68	Hypertension, alcohol abuse, oesophageal varices, diverticulosis, cirrhosis	Native aortic valve	Fever, malaise, weight loss, epistaxis, haematemesis	No	Nil	Amoxicillin and gentamicin for 6 weeks	Survived
Clavero et al. [37]	F/72	Type 2 diabetes mellitus, hypertension, end-stage kidney disease, colonic diverticulosis	Native mitral valve	Fever and chills during haemodialysis	No	Multiorgan failure	Vancomycin and gentamicin	Died
Landeloos et al. [38]	F/82	Atrial fibrillation, hypertension, osteoporosis, history of infective endocarditis with mitral valve replacement, colonic polyp	Prosthetic mitral valve	Fever, anorexia, exertional dyspnoea	Yes (Consumption of fish)	Acute ischaemic stroke of the right occipital lobe	Penicillin for 4 weeks and gentamicin for 2 weeks, followed by amoxicillin for 2 weeks	Survived
Wang et al. [39]	M/72	Mitral valve prolapse, staghorn stone of the right kidney, gastric ulcer	Native mitral valve	Fever and purpuric leg lesions	Yes (Consumption of raw squid)	Nil	Penicillin for 4 weeks and gentamicin for 2 weeks	Survived
Makhoul et al. [13]	M/65	Hypertension, hyperlipidemia, atrial fibrillation, bicuspid aortic valve, ascending aortic aneurysm with a history of a Bentall procedure	Prosthetic aortic valve	Headache, dysarthria and vomiting	No	Left middle cerebral artery aneurysm with haemorrhage	Ceftriaxone and gentamicin for 6 weeks	Survived
Rojas-Velasco et al. [40]	F/60	Chronic rheumatic heart disease with history of mitral, tricuspid and aortic valve replacement, chronic heart failure, diabetes mellitus, hypertension, colonic polyps	Prosthetic aortic valve	Fever	No	Nil	Ceftriaxone	Survived

*L. garvieae* has also been reported to cause infections other than IE, including primary bacteraemia, osteoarticular infections, urinary tract infections, meningitis and intra-abdominal infections (Table 2). Interestingly, *L. garvieae* has been implicated in cases of platelet concentrates contamination, which led to recipient sepsis [[3], [4], [5]]. These case reports suggest the potential role of *L. garvieae* as an emerging pathogen associated with the contamination of blood products and increased vigilance is warranted.

**Table 2:** Published literature on non-infective endocarditis cases caused by *Lactococcus garvieae*.

	Sex/ Age	Comorbid conditions	Clinical syndrome	Site where *L. garvieae* was isolated	Clinical presentation	Fish contact/ consumption	Complications	Treatment	Outcome
Sahu et al. [41]	M/86	Anterior cervical hyperostosis	Bacteraemia	Blood	Lethargy, decreased mentation, hypotension	No	Nil	Ampicillin and Ceftazidime	Survived
Lee et al. [42]	M/77	Diabetes mellitus, hypertension, abdominal aortic aneurysm with endovascular repair	Infective spondylitis	Blood	Back pain	No	No	Cefepime for 1 month then Levofloxacin for 6 months	Survived
Chan et al. [8]	M/70	Gastritis	Infective spondylodiscitis	Bone biopsy of L3-4	Fever and back pain	Yes (Frequent visits to fish market and intake of raw fish)	No	Ampicillin for 6 weeks	Survived
Tandel et al*.* [43]	M/75	Chronic alcoholism	Chronic meningitis	Cerebrospinal fluid	Fever, disorientation, weight loss, fatigue, anorexia	No	No	Ceftriaxone and vancomycin	Survived
Tariq et al. [44]	M/70	Hypertension, benign prostatic hyperplasia, oesophageal cancer with chemoradiotherapy done	Urinary tract infection	Urine	Fever and confusion	No	No	Amoxicillin-clavulanate for 1 week	Survived
Wang et al. [39]	M/10	Oesophageal stricture due to alkaline ingestion with repeated dilatation and subcutaneous colon interposition	Septic shock	Blood	Shock, cyanosis and altered consciousness	Yes (Consumption of grilled Tilapia)	Acute kidney injury, disseminated intravascular coagulation	Not mentioned	Died
Wang et al. [39]	F/56	Small bowel diverticulosis, hypertension, asthma, hyperthyroidism	Small bowel diverticulitis	Blood	Suprapubic pain and fever	No	Nil	Cefazolin and gentamicin for 2 days followed by co-trimoxazole for 5 days	Survived
Wang et al. [39]	M/47	Traffic accident with left globe rupture and multiple facial lacerations requiring surgery	Jejunal perforation	Ascitic fluid	Fever and abdominal pain	Yes (Consumption of sashimi)	Nil	Enterorrhaphy of jejunum, piperacillin and amikacin for 1 week	Survived
Mitra et al. [45]	F/3	Nil	Gastroenteritis	Blood	Fever, anorexia, vomiting, diarrhoea	Yes (Consumption of fish and unpasteurised milk)	Nil	Amoxicillin and metronidazole	Survived
Chao et al. [46]	M/38	IgA nephropathy on peritoneal dialysis	Peritoneal dialysis-associated peritonitis	Peritoneal dialysis effluent	Abdominal pain, diarrhoea, turbid peritoneal dialysis effluent	No	Nil	Intraperitoneal cefazolin for 2 weeks	Survived
Westberg et al. [47]	M/80	Chronic obstructive pulmonary disease, hypertension, hypercholesterolaemia, history of prostate cancer, total hip arthroplasty of the right hip, gastric and duodenal ulcers	Late onset periprosthetic infection of the right hip	Tissue biopsy of the right hip	Right hip and groin pain	No	Nil	Aminopenicillin for 8 weeks	Survived
Choksi et al*.* [48]	M/78	Hypertension, diabetes mellitus, gastroesophageal reflux disease, morbid obesity	Urinary tract infection	Urine	Fever, haematuria, suprapubic tenderness	No	Nil	Broad spectrum antibiotics for 3 days, ceftriaxone for 4 days, cefpodoxime for 7 days	Survived
Neagoe et al. [49]	M/79	Diabetes mellitus, polyarthritis rheumatica, obstructive sleep apnea syndrome, atrial fibrillation with pacemaker implantation, ischaemic heart disease, degenerative disc disease, obesity, antral gastritis, history of endoscopic Zenker's diverticulum repair, history of diverticulitis, right knee replacement due to osteoarthritis	Late onset periprosthetic infection of the right knee	Synovial fluid	Pain, swelling, and motility disorder of the right knee	Yes (Consumption of Nile perch)	Nil	Cefazolin for 24 days, arthroscopic lavage and synovectomy	Survived
Mofredj et al. [50]	F/68	Cholangiocarcinoma treated with biliary stenting and steroid	Liver abscess	Blood	Gastrointestinal bleeding	No	Haemobilia	Removal of biliary stents, amoxicillin, metronidazole and netilmicin	Died
Aubin et al. [51]	F/71	Obesity, hypertension, diabetes mellitus, hemochromatosis, ischaemic cardiomyopathy, chronic alcoholism, osteonecrosis of the femoral head with total hip replacement	Late onset periprosthetic infection of the left hip	Joint fluid and biopsy specimen	Left hip pain	Yes (Working as a fishmonger, frequent consumption of fish and shellfish)	Nil	Two stage exchange arthroplasty, ceftriaxone and levofloxacin for 3 months	Survived
Kim et al. [52]	M/69	Fatty liver, chronic alcoholism with tobacco use, history of gastric ulcer with perforation treated with laparotomy	Acute acalculous cholecystitis	Bile and gallbladder tissue	Upper abdominal pain and postprandial abdominal pain	Yes (Consumption of raw freshwater fish and shellfish)	Nil	Laparoscopic cholecystectomy, cefminox for 3 days and cefaclor for 5 days	Survived
Nakamura et al*.* [4]	M/81	Myelodysplastic syndrome	Bacteraemia from contaminated platelet transfusion	Blood	Fever, hypotension and faecal incontinence after platelet transfusion	No	Nil	Ceftriaxone and meropenem for 1 week	Survived
Nadrah et al*.* [53]	M/81	Congestive heart failure, hypertension, chronic kidney disease, anaemia, pyrophosphate arthritis, diverticulosis of the colon, reflux oesophagitis, hypothyroidism, pacemaker insertion, mitral and aortic valve replacement	Bacteraemia	Blood	Fever, chills, myalgia, headache and malaise	No	Nil	Ampicillin for 6 weeks and gentamicin for 15 days	Survived
Dylewski et al. [54]	M/90	Benign prostatic hypertrophy with transurethral prostate resection done	Urinary tract infection	Urine and blood	Chills and cloudy urine	No	Nil	Amoxicillin-clavulanate for 10 days	Survived
Amarasinghe et al. [55]	M/52	Hypertension, schizophrenia, hepatitis C, chronic obstructive pulmonary disease/ asthma, alcohol abuse	Lumbar epidural abscess	Epidural abscess and blood	Low back pain	No	Nil	Ceftriaxone and Azithromycin for 6 weeks.	Died (due to unrelated causes)
Colagrossi et al. [5]	M/9	Acute lymphoblastic leukaemia	Bacteraemia from contaminated platelet transfusion	Blood	Fever, raised serum inflammatory markers	No	Nil	Tigecycline, Amikacin, Ceftazidime/ avibactam	Survived
Colagrossi et al. [5]	M/4	Acute lymphoblastic leukaemia	Bacteraemia from contaminated platelet transfusion	Blood	Fever, raised serum inflammatory markers	No	Nil	Tigecycline, Amikacin, β-lactams, Quinolones, Metronidazole	Survived
Colagrossi et al*.* [5]	M/9	Acute lymphoblastic leukaemia	Bacteraemia from contaminated platelet transfusion	Blood	Fever, raised serum inflammatory markers	No	Nil	Tigecycline, Gentamicin, Quinolones	Survived
Tessier et al*.* [56]	F/87	Heart failure, end-stage renal failure	Empyema thoracis	Blood, pleural fluid	Coffee-ground emesis, altered mental status, respiratory failure	Yes (Consumption of cooked fish)	Nil	Ceftriaxone, cefepime, metronidazole	Died
Masudi et al*.* [57]	M/6	Nil	Wound infection	Wound	Bilateral leg abrasions with discharge	No	Nil	Cephalexin and topical gentamicin	Survived
McPherson et al. [58]	M/65	Atrial fibrillation, gastrooesophageal reflux disease, history of renal cell carcinoma	Prosthetic knee joint infection	Joint fluid	Pain, warmth and swelling of left knee (6 months after total knee replacement)	No	Nil	Two-stage revision surgery. Ceftriaxone.	Survived

The risk factors associated with *L. garvieae* infection include raw fish contact or consumption and underlying gastrointestinal diseases [6]. Exposure to raw fish was confirmed in one-third of patients with *L. garvieae* infection, among whom, 63.2% (12/19) developed IE. Gastrointestinal disorders were noted in 54.8% (17/31) and 42.3% (11/26) of IE and non-IE patients, respectively. Upper gastrointestinal disorders such as gastroesophageal reflux disease, gastritis or peptic ulcers were the most common, followed by lower gastrointestinal disorders including diverticulosis and colonic polyps. The use of gastric acid suppressants may facilitate the survival of the pathogen. Intestinal mucosal breaches due to polyps, diverticulosis or malignancies predisposes individuals to infection by providing a portal of entry [[6], [7], [8]]. Our patient consumed an assorted platter containing salmon, tuna and yellowtail from a sushi chain prior to the onset of his neurological symptoms. It is possible the patient acquired the infection from the sushi and sashimi in view of the temporal relationship between their consumption and his symptoms. However, a definitive relationship cannot be made as sampling of the food was not possible. Colonoscopy revealed two tubular adenomas which may have predisposed the patient to the infection.

Infectious intracranial aneurysms (IIAs) are most frequently caused by *Staphylococcus aureus* and viridans group streptococci, other bacteria such as coagulase-negative staphylococci, β-haemolytic streptococci, enterococci and Gram-negative bacteria such as the HACEK organisms (*Haemophilus* spp., *Aggregatibacter* spp., *Cardiobacterium hominis, Eikenella corrodens* and *Kingella kingae*) are sometimes implicated, while fungi may also cause IIAs especially in immunocompromised hosts [[9], [10], [11]]. There have been two reported cases of IIA due to *L. garvieae*, but both could be attributed to underlying IE. Watanabe *et al*. described a patient with *L. garvieae* native mitral valve endocarditis causing two IIAs, as well as cerebral and renal infarction [12]. Makhoul et al. reported a patient suffering from *L. garvieae* prosthetic aortic valve IE complicated by mycotic aneurysm in the inferior middle cerebral artery and left frontal haematoma [13]. To our knowledge, this is the first case of an IIA secondary to *L. garvieae* bacteraemia without concomitant IE. The distal PCA location, fusiform nature and histopathology of the aneurysm were highly suggestive of an infected aneurysm, as opposed to the more frequently encountered aneurysms that are generally saccular and located proximally at the circle of Willis.

β-lactam antibiotics such as penicillin and ceftriaxone are the drug of choice for *L. garvieae* infections [7,8,14]. Combination with aminoglycosides can be considered in severe infections to achieve a rapid bactericidal effect [1]. Clindamycin should be avoided as the organism is intrinsically resistant [14]. Neurosurgical or endovascular intervention should be considered in the case of IIAs, in addition to antibiotics.

In conclusion, we report a case of ruptured IIA associated with *L. garvieae* bacteraemia without concomitant IE. In managing invasive infections due to this organism, clinicians should seek history of raw fish exposure and underlying gastrointestinal pathologies. At-risk patients such as those with prosthetic heart valves should be educated to avoid consuming or handling raw fish to reduce the risk of *L. garvieae* infection and its potentially catastrophic complications.

## Funding

This research did not receive any specific grant from funding agencies in the public, commercial, or not-for-profit sectors.

## Author contributions

C.H.L. and P.Y.M.W. contributed to the conception and design of the study. C.H.L. performed the literature search. C.H.L. and P.Y.M.W. analysed the data. C.H.L. and P.Y.M.W. and C.K.L.L. wrote the manuscript. K.S.N. provided the histopathological images. All authors were involved in critical revision of the manuscript and final approval of the manuscript.
